# Supplementary material for: 2D Ultrasound Elasticity Imaging of Abdominal Aortic Aneurysms Using Deep Neural Networks
Source: IEEE Trans Comput Imaging. Author manuscript; Available in PMC 2026 Jun 11. (PMC13251729; doi:10.1109/tci.2026.3694126)
Supplement: supp1-3694126 [file NIHMS2181623-supplement-supp1-3694126.pdf]

Supplementary Materials

Analytical Validation

This section presents the reconstructions for analytically solvable modulus distribution. We used a cross-section of a homogeneous tube that deformed under plane strain conditions to validate the performance of our deep learning (DL) and iterative (ITR) methods [1]. For an isotropic, homogeneous tube subjected to a uniform internal pressure (P_i), and plane strain assumption (where longitudinal strain is 0), the radial displacement at a distance r from the center is given by:

$$u_r = \frac{r}{E(b^2 - a^2)} \left[(P_i a^2 - P_o b^2)(1+\nu)(1-2\nu) - \frac{a^2 b^2 (P_o - P_i)(1+\nu)}{r^2} \right]$$

Here, E is the Young's modulus of 200 kPa (corresponding to a shear modulus of 66.67 kPa), ν is the Poisson's ratio set to 0.5, P_i is the internal lumen pressure of 5.333 kPa, and P_o is the outside pressure set to 0. The inner and outer radii, a and b, are defined as 2.95 cm and 3.45 cm, respectively. The Cartesian displacement components are subsequently calculated as $u_x = u_r \cos(\theta)$ and $u_y = u_r \sin(\theta)$. The derivation of this formula is detailed in [1].

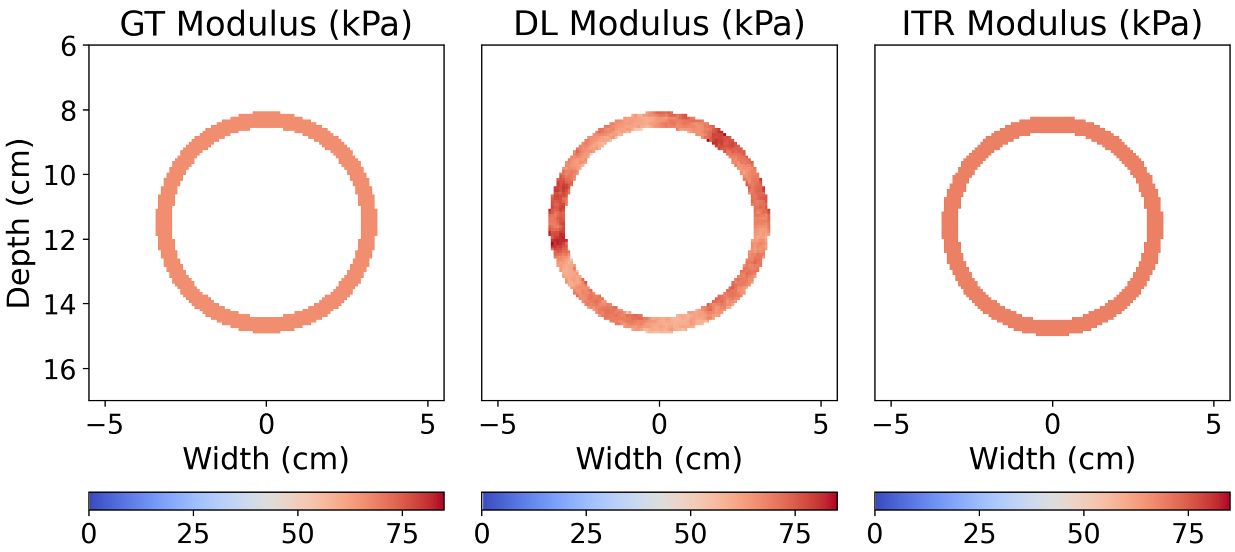

Figure S1: Results for analytical validation of DL and ITR reconstruction methods

These analytically obtained displacements were used as inputs for our deep learning (DL) and iterative (ITR) methods. The resulting modulus predictions are shown in Figure S1. The ground truth modulus had a mean value of 66.67 kPa; the DL method and the ITR method yielded a mean modulus of 67.85 ± 5.79 kPa and 68.59 ± 0.02 kPa, respectively. The close agreement between the overall magnitude of the predictions and the ground truth modulus demonstrates the accuracy and reliability of these methods in recovering the modulus magnitude.

Error Image

To assess the reconstruction accuracy, error images were computed using the equation:

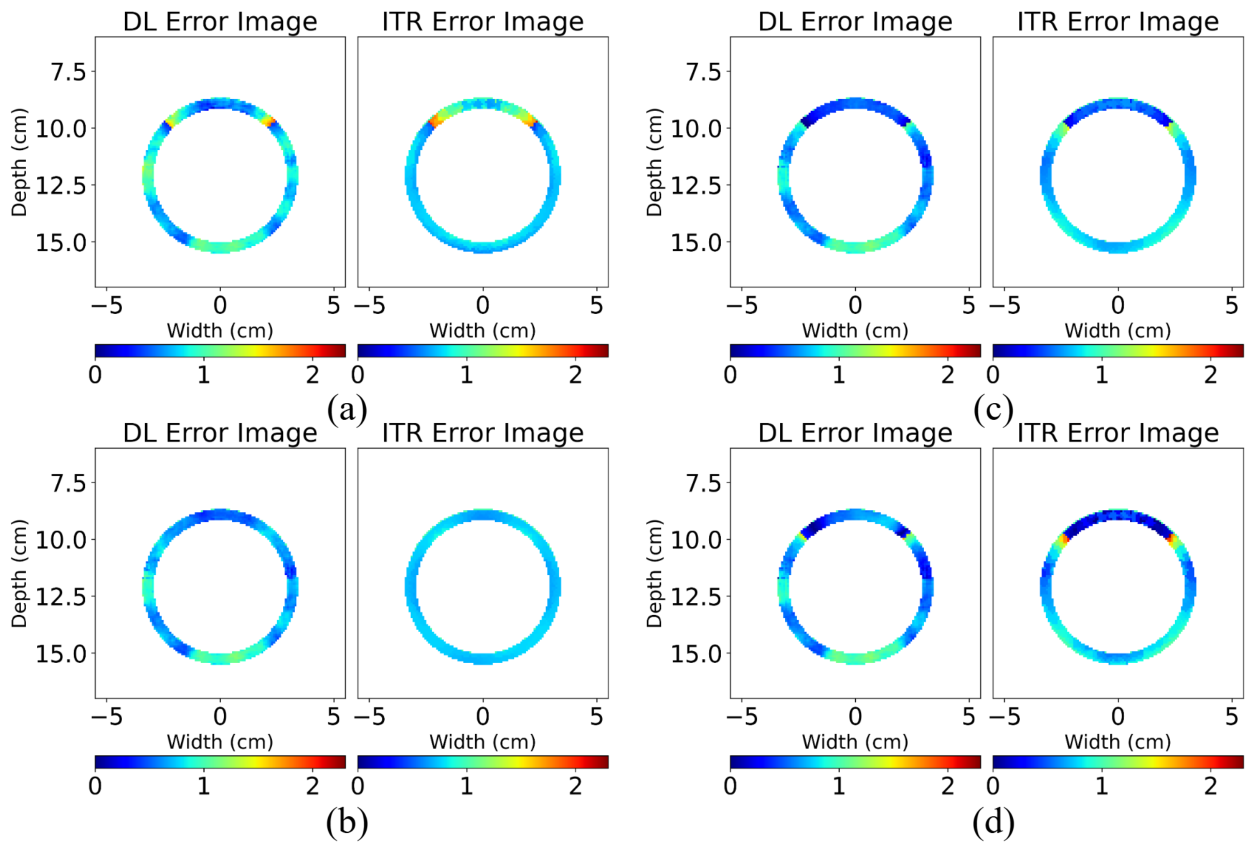

Figure S2: COMSOL error images for (a) COMSOL model 1, (b) COMSOL model 2, (c) COMSOL model 3, and (d) COMSOL model 4.

$$\text{Error}\,\langle A, B\,\rangle := \frac{|A - B|}{|A|}, \tag{1}$$

where A is the ground truth (GT) modulus image, B is the predicted modulus image, obtained either from the deep learning (DL) method or the iterative (ITR) method. The error images are shown in Figure S2.

References

[1] C. de Korte, *Intravascular ultrasound elastography*. 1999.
